# Associations Between Intellectual and Social Activities With Frailty Among Community-Dwelling Older Adults in China: A Prospective Cohort Study

**DOI:** 10.3389/fmed.2021.693818

**Published:** 2021-07-26

**Authors:** Yafang Huang, Xiangyu Guo, Juan Du, Yanli Liu

**Affiliations:** ^1^School of General Practice and Continuing Education, Capital Medical University, Beijing, China; ^2^School of Pharmaceutical Sciences, Capital Medical University, Beijing, China; ^3^Department of General Practice, Beijing Tiantan Hospital, Capital Medical University, Beijing, China

**Keywords:** frailty, intellectual activity, social activity, prospective cohort study, CHARLS

## Abstract

**Background:** Frailty is one of the most important global health challenges. We aimed to examine the associations between frequency of intellectual and social activities and frailty among community-dwelling older adults in China.

**Methods:** This is a prospective analysis of older adults (aged ≥60 years) who had intellectual and social activity data and were free of frailty from the national representative China Health and Retirement Longitudinal Study (CHARLS). The exposure was frequency of intellectual and social activities. Frailty was measured by the frailty index (FI) and defined as FI ≥ 0.25. Frailty incidents were followed up for 2 years. We estimated the relative risks (RRs) with 95% confidence intervals (CIs) using log-linear binominal regression adjusting for potential confounders.

**Results:** We documented 655 frailty cases over the past 2 years. Participants who had frequent intellectual activities had a lower frailty risk compared with participants who did not have intellectual activity (adjusted RR = 0.65, 95%CI = 0.47–0.90). The adjusted RRs were 0.51 (95%CI = 0.33–0.77) for participants who did not have a slip or a fall accident and 1.06 (95%CI = 0.65–1.75) for participants who had experienced slip and fall accidents (*P* = 0.01 for interaction). Having frequent social activities was not associated with a significant decrease in frailty risk compared with participants who did not have social activity (adjusted RR = 0.93, 95%CI = 0.78–1.12).

**Conclusions:** This observational study showed that having frequent intellectual activities was associated with a decreased frailty risk. The association was likely to be stronger in participants without a slip or a fall accident. Randomized controlled trials are needed to confirm this observational finding.

## Introduction

Frailty, as an extreme consequence of the normal aging process, is one of the most serious global health challenges (1). A recent systematic review and meta-analysis has reported that the pooled prevalence of frailty was 17.4% among community-dwelling older adults in low-income and middle-income countries (2). Frailty is an unstable status in which the physiological reserves are reduced, causing disorders in homeostatic systems (1, 3). This would lead to rapid deterioration in functional capacity across many physiological systems and, thus, significantly increased risks of adverse health outcomes, such as falls, disability, hospitalization, and death (1, 3). Therefore, the identification of and interventions to slow the progression of frailty are essential for healthcare systems in an aging society (4, 5).

Insights into the key risk factors of frailty would be very helpful in determining effective strategies for frailty prevention. Many cross-sectional and longitudinal studies have been conducted to explore factors associated with frailty (2, 6–22). The identified potential factors included sociodemographic factors (6–9), socioeconomic status (2, 7, 14, 15), physical and biological factors (20–22), and lifestyle and clinical factors (7, 11–14, 18, 19). Most of these risk factors could be modified by regular physical activities and adequate nutritional intake (23).

Several studies have shown that participation in social and intellectual activities could improve the cognitive reserve and reduce functional decline and disabilities (12, 23, 24). Social and intellectual activities, along with physical activities and nutritional intake, play an important role in frailty prevention (2, 12). The associations between social or intellectual activities and physical frailty have been investigated in many studies (25–32). For example, a 4-year cohort study in Japan found that social frailty was a significant risk factor that leads to physical frailty (25). Another study in Japan showed that social activities decreased the functional disability risks (32). Wang et al. conducted a cross-sectional study among seniors from Singapore (28). They found that participation in intellectual activities was likely to be associated with a lower frailty prevalence (28).

To date, prospective cohort evidence for the associations between intellectual and social activities and frailty is still lacking in China. In addition, the associations between the different frequencies of intellectual or social activity participation and frailty development also needed to be further investigated (3, 33–35). We therefore conducted this prospective study to evaluate the associations between the frequency of intellectual and social activities and frailty among Chinese community-dwelling older adults.

## Methods

### Study Population and Design

The analyses were performed based on the China Health and Retirement Longitudinal Study (CHARLS) (36). In brief, CHARLS is a biennial national study that collects a representative sample of Chinese residents using a multistage stratified probability proportionate to size technique. High-quality information of the included residents was collected. The details of the objectives and methods of CHARLS were published in a previous report (36). The survey in 2015–2016 and the follow-up survey in 2017–2018 were available for the analyses in this study. The CHARLS was approved by the Biomedical Ethics Review Committee of Peking University. Written informed consent was obtained from all participants. The ethical approval number of CHARLS is IRB00001052-11015.

**Figure 1:**
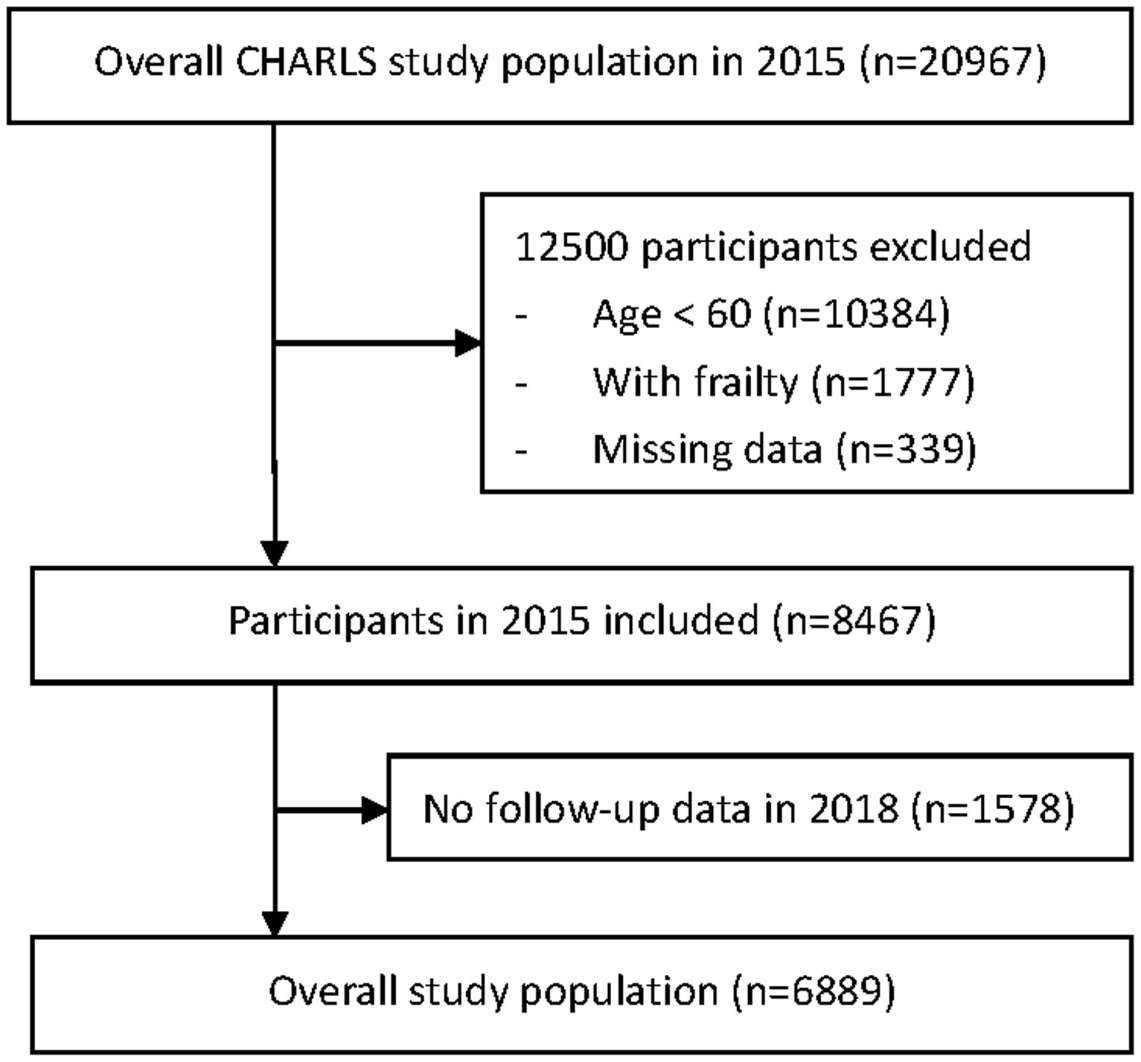
Flowchart of participant selection from the China Health and Retirement Longitudinal Study (CHARLS).

For the current analysis, we restricted the participants to those aged 60 years or above. We also excluded participants without frailty information. For each participant, the intellectual and social performances were collected in 2015–2016. Each participant had a 2-year follow-up. The ascertainment of frailty was carried out in 2017–2018. The participants who did not respond to the 2018 survey were considered as lost to follow-up (see Figure 1). The data in this study were reported according to the Strengthening the Reporting of Observational Studies in Epidemiology (STROBE) reporting guidelines (37).

### Assessment of the Frequencies of Intellectual and Social Activities

In the 2015–2016 CHARLS survey, participants were asked about their intellectual and social activities in the past month. Intellectual activities include playing Ma-jong, chess, cards; attending an educational or training course; investing in stock; and surfing the Internet. Social activities include interacting with friends; going to a sport, social, or other kinds of club; taking part in a community-related organization; and doing voluntary or charity work. The frequency of each activity was rated as follows: almost daily (score = 3), almost every week (score = 2), not regularly (score = 1), or never (score = 0). The total scores for intellectual and social activities ranged from 12 to 0 points and were categorized as ≥3, 1–2, and 0, where “≥3” referred to frequent participation, “1-2” referred to non-regular participation, and “0” referred to no participation in intellectual and social activities (30).

### Ascertainment of Frailty

Frailty was measured by using a frailty index (FI). The construction of the FI was based on a standard procedure (38). The detailed method for the calculation of FI was reported in previous published studies (13, 17). In brief, a total of 39 deficit variables that were associated with FI in the CHARLS were selected, including 15 comorbidity variables, 5 disability variables, and 19 variables on activities of daily living. All of these 39 variables were scored from 0 to 1, where “0” indicated no deficit and “1” indicated the presence of a deficit. For each participant, FI was calculated by adding the scores of all the deficits and dividing by the total number of deficits. Frailty was defined as FI ≥ 0.25 (13, 16, 17, 39, 40).

### Assessment of Covariates

The following information were obtained: sociodemographic factors, including age and sex; socioeconomic factors, including economic development regions (>$10,000, from $10,000 to >7,000, and ≤ $7,000) (41); lifestyle and health factors, including hours of actual sleep (≥6 h or <6 h), smoking (yes or no), and whether one had experienced slip and fall accidents (yes or no). The participants were deemed to have slip and fall accidents if they responded “yes” when asked “Have you fallen down?” The main comorbidities in medical history included cancer, diabetes mellitus, heart disease, hypertension, and stroke.

### Statistical Analysis

Baseline characteristics were compared among the different intellectual and social activity scores by using one-way analysis of variance (ANOVA) for continuous measures and using the Mantel–Haenszel test for proportion trends. The associations between intellectual and social activities and frailty were estimated as relative risks (RRs) with 95% confidence intervals (CIs) using log-linear binominal regression with a multivariable-adjusted model. In the multivariable-adjusted model 1, we adjusted for age and sex. To control potential confounding from socioeconomic status, we additionally adjusted for economic development regions in the multivariable-adjusted model 2. In the multivariable-adjusted model 3, we additionally controlled for lifestyle and health factors, such as sleep, smoking, and experiences of slip and fall accidents. Moreover, we finally introduced a model 4 to additionally adjust for the main comorbidities such as cancer, diabetes mellitus, heart disease, hypertension, and stroke. A multivariate logistic regression analysis was used in model 4. Odd ratios (ORs) with 95% CIs were reported.

Based on a review of previous literature (8, 16, 17), whether one had experienced slip and fall accidents was a potential effect modifier that may modify the associations between intellectual and social activities and frailty. Therefore, subgroup analysis was conducted based on whether the participants had experienced slip and fall accidents.

A number of sensitivity analyses were performed to examine the robustness of the associations between intellectual and social activities and frailty. Firstly, the impacts of lowering the cutoff value of FI were estimated, as FI ≥ 0.24 and FI ≥ 0.23. Secondly, only participants aged 65 years or older were included. Thirdly, we included only the participants without missing data. A *p*-value <0.05 was considered significant. All the analyses were performed using SAS, version 9.4 (SAS Institute, Cary, NC, USA).

## Results

This study included 6,889 participants. Table 1 presents the baseline characteristics according to the intellectual activity and social activity scores. Of the sample, 78.3% of the participants had an intellectual activity score of 0 and 62.4% had a social activity score of 0. Among the different intellectual activity scores, there were differences in the baseline characteristics such as age, sex, economic development region, actual sleep, smoking, cancer, diabetes, and heart disease. Among the different social activity scores, there were differences in the baseline characteristics such as age, sex, slip and fall accidents, smoking, diabetes, heart disease, and hypertension.

**Table 1:** Baseline characteristics according to the intellectual activity and social activity scores.

	**Intellectual activity scores**				**Social activity scores**			
	**0**	**1–2**	**≥3**	***P*-value^**a**^**	**0**	**1–2**	**≥3**	***P*-value^**a**^**
No. of participants	5,395	848	646		4,302	1,147	1,440	
Age, mean (SD) (years)	67.4 (6.1)	66.4 (5.5)	67.0 (5.8)	<0.0001	67.2 (5.9)	66.9 (5.8)	67.7 (6.3)	0.0023
Male, *n* (%)	2547 (47.2)	533 (62.9)	403 (62.4)	<0.0001	2,273 (52.8)	594 (51.8)	616 (42.8)	<0.0001
Economic development region, *n* (%)				0.0084				0.5143
>$10,000	1,883 (34.9)	294 (34.8)	235 (36.4)		1,508 (35.1)	418 (36.5)	486 (33.8)	
$10,000 to > 7,000	2,487 (46.2)	439 (51.9)	319 (49.4)		2,038 (47.4)	511 (44.6)	696 (48.4)	
≤ $7,000	1,019 (18.9)	113 (13.4)	92 (14.2)		752 (17.5)	217 (18.9)	255 (17.8)	
Actual sleep <6 h, *n* (%)	1,974 (36.6)	264 (31.1)	188 (29.1)	<0.0001	1,556 (36.2)	378 (33.0)	492 (34.2)	0.0816
Without falling down experience, *n* (%)	4,395 (81.6)	704 (83.0)	541 (83.8)	0.1114	3,565 (83.0)	926 (80.7)	1,149 (79.8)	0.0033
Never smoker, *n* (%)	3,032 (58.0)	337 (41.0)	276 (44.0)	<0.0001	2,208 (53.0)	587 (52.4)	850 (60.8)	<0.0001
**Medical history**, ***n*** **(%)**								
Cancer	87 (1.6)	21 (2.5)	18 (2.8)	0.0112	69 (1.6)	24 (2.1)	33 (2.3)	0.0728
Diabetes mellitus	606 (11.5)	107 (12.9)	117 (18.6)	<0.0001	469 (11.2)	157 (14.0)	204 (14.5)	0.0003
Heart disease	1,014 (19.6)	191 (23.6)	151 (25.2)	0.0001	774 (18.8)	250 (22.7)	332 (24.5)	<0.0001
Hypertension	2,146 (42.4)	321 (40.2)	274 (45.1)	0.5427	1,687 (41.8)	428 (39.5)	626 (46.4)	0.0154
Stroke	385 (7.2)	48 (5.8)	58 (9.2)	0.3467	297 (7.0)	69 (6.1)	125 (8.8)	0.0621

a *Mantel–Haenszel test for proportion trends and one-way ANOVA for continuous measures*.

We observed 655 frailty cases over the past 2 years. In the fully adjusted model (model 3), participants with frequent intellectual activities (score ≥ 3) had a lower frailty risk compared with participants who did not have intellectual activity (scores = 0), with multivariable-adjusted RR of 0.65 (95%CI = 0.47–0.90). Participants who had non-regular intellectual activities (score = 1–2) had a lower frailty risk compared with participants who did not have intellectual activity, with multivariable-adjusted RR of 0.60 (95%CI = 0.44–0.80).

Results from the adjusted model (model 3) showed that participants who had non-regular social activities (score = 1–2) had a lower frailty risk compared with participants who had no social activity (score = 0), with multivariable-adjusted RR of 0.68 (95%CI = 0.54–0.86). However, having frequent social activities (score ≥ 3) was not associated with a decreased frailty risk compared with participants who had no social activity (score = 0), with multivariable-adjusted RR of 0.93 (95%CI = 0.78–1.12) (Table 2).

**Table 2:** Risk of frailty according to the intellectual activity and social activity scores.

**Scores**	**No. of cases**	**Multivariable-adjusted model 1**		**Multivariable-adjusted model 2**		**Multivariable-adjusted model 3**		**Multivariable-adjusted model 4**	
		**Relative risk (95%CI)**	***P*-value**	**Relative risk (95%CI)**	***P*-value**	**Relative risk (95%CI)**	***P*-value**	**Odds ratio (95%CI)**	***P*-value**
**Intellectual activity scores**									
0	567/5,395	1.00 (Reference)	**–**	1.00 (Reference)	**–**	1.00 (Reference)	**–**	1.00 (Reference)	**–**
1–2	48/848	0.64 (0.48–0.86)	0.0027	0.64 (0.48–0.86)	0.0026	0.60 (0.44–0.80)	0.0007	0.51 (0.35–0.73)	0.0003
≥3	40/646	0.67 (0.49–0.92)	0.0119	0.67 (0.49–0.91)	0.0116	0.65 (0.47–0.90)	0.0092	0.40 (0.26–0.61)	<0.0001
**Social activity scores**									
0	441/4,302	1.00 (Reference)	**–**	1.00 (Reference)	**–**	1.00 (Reference)	**–**	1.00 (Reference)	**–**
1–2	77/1,147	0.70 (0.55–0.88)	0.0024	0.70 (0.56–0.89)	0.0032	0.68 (0.54–0.86)	0.0016	0.57 (0.42–0.77)	0.0003
≥3	137/1,440	0.92 (0.76–1.10)	0.3604	0.92 (0.76–1.10)	0.3615	0.93 (0.78–1.12)	0.4654	0.93 (0.73–1.18)	0.5453

*Multivariable-adjusted model 1: adjusted for age and sex*.

*Multivariable-adjusted model 2: additionally adjusted for economic development regions (>$10,000, $10,000 to >7,000, and ≤ $7,000)*.

*Multivariable-adjusted model 3: additionally adjusted for sleep (≥6 h or <6 h), smoking (never, past, or current), and whether one had a fall experience (yes or no)*.

*Multivariable-adjusted model 4: additionally adjusted for cancer, diabetes mellitus, heart disease, hypertension, and stroke. Multivariate logistic regression analyses were used in model 4*.

Subgroup analysis was conducted to detect whether having experienced slip and fall accidents was an interaction that modified the effect of intellectual activity on frailty. We found evidence of an interaction effect of “slip and fall accidents” when comparing participants who had frequent intellectual activities (score ≥ 3) to those who did not have intellectual activity (score = 0). Among the participants who did not have a slip or a fall accident, having frequent intellectual activities (score ≥ 3) was associated with a significant decrease in frailty risk compared with participants who did not have intellectual activity (score = 0). However, among the participants who had experienced slip and fall accidents, having frequent intellectual activities (score ≥ 3) was not associated with a decreased frailty risk compared with participants who had no intellectual activity (score = 0). The *p*-value for the “slip and fall accidents” interaction was 0.0103 (Table 3).

**Table 3:** Subgroup analyses of the intellectual activity and social activity scores and the risk of frailty.

**Scores**	**Slip and fall experience**	**Relative risk (95%CI)**	***P* interaction^**a**^**
**Intellectual activity scores**			
1–2 vs. 0	Yes	0.72 (0.43–1.18)	0.3511
	No	0.56 (0.38–0.81)	
≥3 vs. 0	Yes	1.06 (0.65–1.75)	0.0103
	No	0.51 (0.33–0.77)	
**Social activity scores**			
1–2 vs. 0	Yes	0.81 (0.54–1.22)	0.2918
	No	0.64 (0.48–0.86)	
≥3 vs. 0	Yes	1.04 (0.75–1.45)	0.1983
	No	0.88 (0.70–1.10)	

a *Estimated effects were adjusted on the fully adjusted model 3 (see footnote in Table 2)*.

In the sensitivity analyses, all the results were generally unchanged, indicating the robustness of the identified associations (Table 4).

**Table 4:** Sensitivity analyses: risk of frailty according to the intellectual activity and social activity scores.

		**No. of cases**	**Multivariable-adjusted model 1**		**Multivariable-adjusted model 2**		**Multivariable-adjusted model 3**		**Multivariable-adjusted model 4**	
			**Relative risk (95%CI)**	***P*-value**	**Relative risk (95%CI)**	***P*-value**	**Relative risk (95%CI)**	***P*-value**	**Odds ratio (95%CI)**	***P*-value**
SA1	**Intellectual activity scores**									
	0	653/5,395	1.00 (Reference)	**–**	1.00 (Reference)	**–**	1.00 (Reference)	**–**	1.00 (Reference)	**–**
	1–2	57/848	0.65 (0.50–0.85)	0.0016	0.65 (0.50–0.85)	0.0015	0.62 (0.47–0.81)	0.0005	0.54 (0.38–0.75)	0.0003
	≥3	46/646	0.66 (0.50–0.89)	0.0058	0.66 (0.50–0.89)	0.0056	0.65 (0.48–0.88)	0.0050	0.40 (0.27–0.59)	<0.0001
	**Social activity scores**									
	0	502/4,302	1.00 (Reference)	**–**	1.00 (Reference)	**–**	1.00 (Reference)	**–**	1.00 (Reference)	**–**
	1–2	93/1,147	0.74 (0.60–0.91)	0.0053	0.75 (0.61–0.92)	0.0067	0.72 (0.58–0.90)	0.0030	0.59 (0.45–0.79)	0.0003
	≥3	161/1,440	0.95 (0.80–1.12)	0.5394	0.95 (0.80–1.12)	0.5458	0.96 (0.81–1.14)	0.6601	0.97 (0.77–1.22)	0.7917
SA2	**Intellectual activity scores**									
	0	731/5,395	1.00 (Reference)	**–**	1.00 (Reference)	**–**	1.00 (Reference)	**–**	1.00 (Reference)	**–**
	1–2	69/848	0.70 (0.56–0.89)	0.0039	0.70 (0.55–0.89)	0.0038	0.67 (0.52–0.85)	0.0013	0.58 (0.42–0.79)	0.0006
	≥3	56/646	0.72 (0.56–0.94)	0.0147	0.72 (0.56–0.94)	0.0147	0.72 (0.55–0.94)	0.0164	0.47 (0.33–0.68)	<0.0001
	**Social activity scores**									
	0	564/4,302	1.00 (Reference)	**–**	1.00 (Reference)	**–**	1.00 (Reference)	**–**	1.00 (Reference)	**–**
	1–2	110/1,147	0.77 (0.64–0.94)	0.0086	0.78 (0.64–0.94)	0.0110	0.76 (0.63–0.93)	0.0069	0.64 (0.49–0.84)	0.0011
	≥3	182/1,440	0.94 (0.81–1.10)	0.4715	0.94 (0.81–1.10)	0.4691	0.95 (0.81–1.11)	0.5145	0.95 (0.77–1.19)	0.6732
SA3	**Intellectual activity scores**									
	0	539/4,953	1.00 (Reference)	**–**	1.00 (Reference)	**–**	1.00 (Reference)	**–**	1.00 (Reference)	**–**
	1–2	45/760	0.65 (0.48–0.87)	0.0042	0.65 (0.48–0.87)	0.0039	0.61 (0.45–0.83)	0.0015	0.51 (0.35–0.75)	0.0005
	≥3	38/588	0.68 (0.49–0.94)	0.0178	0.68 (0.49–0.94)	0.0180	0.65 (0.47–0.91)	0.0125	0.39 (0.25–0.60)	<0.0001
	**Social activity scores**									
	0	419/3,939	1.00 (Reference)	**–**	1.00 (Reference)	**–**	1.00 (Reference)	**–**	1.00 (Reference)	**–**
	1–2	74/1,040	0.71 (0.56–0.90)	0.0044	0.72 (0.57–0.91)	0.0058	0.69 (0.54–0.88)	0.0025	0.57 (0.42–0.78)	0.0005
	≥3	129/1,322	0.90 (0.75–1.09)	0.2952	0.90 (0.75–1.09)	0.2902	0.92 (0.76–1.11)	0.4011	0.92 (0.72–1.18)	0.5217
SA4	**Intellectual activity scores**									
	0	549/5,226	1.00 (Reference)	**–**	1.00 (Reference)	**–**	1.00 (Reference)	**–**	1.00 (Reference)	**–**
	1–2	44/820	0.61 (0.45–0.83)	0.0014	0.61 (0.45–0.83)	0.0014	0.60 (0.44–0.80)	0.0007	0.51 (0.35–0.73)	0.0003
	≥3	37/628	0.64 (0.46–0.88)	0.0062	0.64 (0.46–0.88)	0.0063	0.65 (0.47–0.90)	0.0092	0.40 (0.26–0.61)	<0.0001
	**Social activity scores**									
	0	425/4,161	1.00 (Reference)	**–**	1.00 (Reference)	**–**	1.00 (Reference)	**–**	1.00 (Reference)	**–**
	1–2	72/1,119	0.68 (0.53–0.86)	0.0016	0.68 (0.54–0.87)	0.0019	0.68 (0.54–0.86)	0.0016	0.57 (0.42–0.77)	0.0003
	≥3	133/1,394	0.94 (0.78–1.13)	0.4816	0.93 (0.77–1.12)	0.4366	0.93 (0.78–1.12)	0.4654	0.93 (0.73–1.18)	0.5453

*Multivariable-adjusted model 1: adjusted for age and sex*.

*Multivariable-adjusted model 2: additionally adjusted for economic development regions (>$10,000, $10,000 to >7,000, and ≤ $7,000)*.

*Multivariable-adjusted model 3: additionally adjusted for sleep (≥6 h or <6 h), smoking (never, past, or current), and whether one had a fall down experience (yes or no)*.

*Multivariable-adjusted model 4: additionally adjusted for cancer, diabetes mellitus, heart disease, hypertension, and stroke. Multivariate logistic regression analyses were used in model 4*.

*SA1: defined frailty as FI ≥ 0.24 in sensitivity analysis 1*.

*SA2: defined frailty as FI ≥ 0.23 in sensitivity analysis 2*.

*SA3: included only those participants ≥65 years old in sensitivity analysis 3*.

*SA4: included only the participants without missing data in sensitivity analysis 4*.

*SA, sensitivity analysis*.

## Discussion

In this prospective analysis of 6,889 elderly Chinese participants, 655 frailty cases were identified over the 2-year follow-up. We found that having frequent intellectual activities was associated with a 35% lower risk of frailty. The association was likely to be stronger among participants who did not experience a slip or a fall accident, with a 49% lower risk of frailty. These associations showed robustness in a series of sensitivity analyses. On the contrary, having frequent social activities was not associated with a significant decrease in frailty risk compared with participants who did not have social activity.

Previous studies have shown that risk factors for the onset of frailty span across a broad range, including sociodemographic, socioeconomic, lifestyle-related, and biological and clinical aspects (2, 6–22). Feng et al. conducted a systematic review and meta-analysis to investigate protective factors that were associated with frailty among elderly people (7). A wider range of factors was identified, including psychological and social factors (7). Our study is in agreement with these previous findings.

The identification of essential modifiable risk and protective factors is very important for frailty prevention (1, 3, 42). Previously, the preventive strategies mainly focused on physical-related interventions, such as taking regular physical activities and providing adequate nutritional intake (4, 33, 43). Recently, loneliness and social isolation have been proven to have negative effects on health (10, 44, 45). More attention should be paid to the association between psychosocial factors and frailty development. In a 4-year cohort study, Makizako et al. found that social frailty leads to physical frailty in a relatively short period of time (25). Based on a 2-year cohort, the results from the study of Ye et al. showed that social participation was associated with a higher prefrail improvement (29). Kim et al. conducted a cross-sectional study to investigate the frequency of social activity participation and its association with the different levels of frailty (27). They found that social activities such as leisure and club activities at a frequency of once a week were associated with frailty prevention (27). The results of this study were in agreement with these previous findings, despite the differences in the setting population, the frailty index domains, and details in the social and intellectual activities included between this study and the previous studies. In addition, we found that non-regular participation in social activities has a positive impact on frailty prevention. However, having frequent social activities (such as an almost daily participation) was not associated with a decrease in frailty risk.

Understanding the associations between intellectual activity participation and frailty development was also important. In a cross-sectional study, Wang et al. found that engaging in intellectual activities in late-life was associated with a lower frailty prevalence, especially among female elderly people (28). To date, evidence from prospective cohort studies is still lacking for the impact of intellectual activities on frailty. The present study showed that participation in intellectual activity was associated with a significant decrease in frailty risk compared with non-participation in intellectual activity. Frailty often coexists with cognitive impairment (45, 46). Lack of intellectual activity increases the risks of cognitive impairment (30, 47, 48). In the future, strategies for frailty prevention should be more focused on improving participation in intellectual activities. In addition, intellectual training, when combined with physical training, could have a positive effect on preserving the function of physiological systems and, thus, slowing the progression of cognitive frailty (12, 48, 49), despite the underlying biological and psychological mechanisms still far from being understood (1, 48, 50).

Effective strategies are needed to prevent or slow the progression of frailty. To date, solid evidence, such as randomized controlled trials (RCTs), is still lacking to evaluate the effectiveness of intervention strategies on frailty development. Most of the previous studies were observational. They were mainly focused on physical activity and nutritional strategies, such as exercise and muscle training and sufficient protein intake (1, 4, 5, 51). Since the current observational evidence showed a significant association between participation in intellectual activities and decreased frailty risk, it is encouraged to include intellectual activities in the intervention strategies on frailty in the future. Individually tailored intellectual and social activity programs could be added into traditional frailty intervention strategies. Moreover, high-quality RCTs are also needed to examine the effectiveness of these intellectual activity programs on frailty prevention.

## Strengths and Limitations of this Study

To our knowledge, this is the first prospective observational study investigating the associations between intellectual and social activities and frailty risks. We identified that having frequent intellectual activities is associated with decreased frailty risks whereas having frequent social activities is not compared with non-participation in intellectual and social activities. In addition, fall was a significant interaction for the effect of intellectual activity on frailty. The findings in this study would provide useful evidence for the management of and prevention strategies on frailty.

However, two potential limitations should be noted. Firstly, the current research was an observational cohort study. Despite potential confounders being adjusted in the log-linear binominal regression by multivariable-adjusted models, the results may still be biased by other potential important confounders, for example, nutrient intake, musculoskeletal function, and laboratory parameters such as serum uric acid levels (7). On the one hand, the analyses in this study were based on secondary data, so important factors such as nutrition and exercise were precluded. On the other hand, due to model limitation, the number of cases was too small to include enough adjusted variables in the adjusted model. In the future, RCTs are needed to determine the effect of the different levels of intellectual and social activities on frailty. Then, both the known and unknown confounders would be controlled in well-designed RCTs. Secondly, frailty should be detected reliably. Although multiple screening instruments for frailty have been developed and validated, to date, there is still a lack of the most effective instruments to detect frailty. There is also a lack of agreement between the different screening instruments. Nevertheless, in this study, multiple sensitivity analyses with different cutoff values of the FI were performed and the results were robust.

## Conclusions

Overall, this prospective analysis study showed that having frequent intellectual activities was associated with a decreased risk of frailty, particularly in those participants who did not have slip and fall accidents. Non-regular participation in social activities was associated with a decreased risk of frailty compared with no social activity, whereas frequent social activity participation was not. These conclusions were based on observational evidences. In the future, more well-designed cohort studies and RCTs are still required to confirm our findings.

## Data Availability Statement

The datasets presented in this study can be found in online repositories. The names of the repository/repositories and accession number(s) can be found at: This analysis uses data or information from the Harmonized China Health and Retirement Longitudinal Study (CHARLS) dataset and Codebook, version C, which was developed by the Gateway to Global Aging Data (https://g2aging.org). The development of the Harmonized CHARLS was funded by the National Institute on Aging (grants R01 AG030153, RC2 AG036619, and R03 AG043052). Further inquiries can be directed to the corresponding authors. Data can also be obtained on request (yafang@ccmu.edu.cn; xyguo@ccmu.edu.cn).

## Ethics Statement

The studies involving human participants were reviewed and approved by Biomedical Ethics Review Committee of Peking University. The patients/participants provided their written informed consent to participate in this study. Written informed consent was obtained from the individual(s) for the publication of any potentially identifiable images or data included in this article.

## Author Contributions

XG and YH contributed to conception of the study and drafted the manuscript. YH, XG, JD, and YL helped with acquisition, analysis, or interpretation of data. YH, XG, and JD critically revised the manuscript for important intellectual content. YH, XG, and YL performed statistical analysis. XG and YH provided administrative, technical, or material support, had full access to all of the data in the study and take responsibility for the integrity of the data and the accuracy of the data analysis. All authors contributed to the article and approved the submitted version.

## Conflict of Interest

The authors declare that the research was conducted in the absence of any commercial or financial relationships that could be construed as a potential conflict of interest.

## Publisher's Note

All claims expressed in this article are solely those of the authors and do not necessarily represent those of their affiliated organizations, or those of the publisher, the editors and the reviewers. Any product that may be evaluated in this article, or claim that may be made by its manufacturer, is not guaranteed or endorsed by the publisher.
